# Antimildew Effect of Three Phenolic Compounds and the Efficacy of Antimildew Sliced Bamboo Veneer

**DOI:** 10.3390/molecules28134941

**Published:** 2023-06-23

**Authors:** Shiqin Chen, Yingying Shan, Chunlin Liu, Chungui Du, Jiawei Zhu, Fei Yang, Yuran Shao, Qichao Bao, Yuting Wang, Ying Ran, Wenxiu Yin

**Affiliations:** College of Chemistry and Materials Engineering, Zhejiang A&F University, Hangzhou 311300, China; 18768107239@163.com (S.C.); syy15968566686@163.com (Y.S.); eustaceweaver7187@gmail.com (C.L.); yangfeier0826@163.com (F.Y.); shao18309819091@163.com (Y.S.); bqc1125573308@gmail.com (Q.B.); wangyuting270229@icloud.com (Y.W.); ry18119295807@163.com (Y.R.); yinwenxiu_110@163.com (W.Y.)

**Keywords:** Chinese herbal phenolic compounds, sliced bamboo veneer, mildews, antimildew effect

## Abstract

The development of the bamboo industry has been hindered by environmental issues caused by the application of bamboo preservatives. Chinese herbal phenolic compounds have been shown to possess broad-spectrum, potent antimildew properties, making them promising candidates for the development of new bamboo mildew inhibitors. In this study, we investigated the antimildew properties of three phenolic compounds, eugenol, carvacrol, and paeonol, against common mildews in bamboo materials using the Oxford cup method and the double-dilution method. Scanning electron microscopy (SEM) and transmission electron microscopy (TEM) were used to analyze the antimildew mechanism and its effects on mildew cell morphology. Our results showed that carvacrol exhibited the strongest antimildew activity, with minimum inhibitory concentration (MIC) and minimum fungicidal concentration (MFC) values of 1.56 mg/mL and 1.76 mg/mL, respectively, followed by eugenol and paeonol. At a concentration of 25 mg/mL, eugenol and carvacrol had an inhibitory rate of over 50% against various mildews. Different concentrations of the three compounds significantly disrupted the morphology and structural integrity of mildew hyphae, with the extent of damage increasing with concentration and treatment duration. In the sliced bamboo mildew prevention experiment, carvacrol at a concentration of 29.25 mg/mL was found to be highly effective against all tested mildews. Our study provides new insights and a theoretical basis for the development of eco-friendly bamboo mildew inhibitors based on plant phenolic compounds.

## 1. Introduction

Bamboo materials are among the world’s most well-known fast-growing, and high-yielding plants, with short maturation periods, high yields, straight and dry shapes, and dense textures. They are widely used in various fields, such as furniture, wood-based panels, papermaking, and outdoor building materials [1,2,3,4]. As natural reinforcement material in concrete, bamboo has expanded its market and alleviated the supply–demand contradiction of wood resources [5]. However, the long-term mildew prevention issue for outdoor bamboo products remains unresolved [6], resulting in the service life of existing mildew-resistant bamboo products under outdoor conditions rarely exceeding one year. At present, approximately 10% of the total annual bamboo production worldwide is discarded due to mildew, limiting its application scope and causing significant resource waste and economic loss. Although traditional chemical mildew inhibitors are effective [7,8,9], their high toxicity and residual levels pose fatal risks to the environment and humans. Therefore, the search for high-efficiency, green, and eco-friendly mildew inhibitors is of utmost importance for the future application of bamboo materials.

Chinese herbal medicines, as natural products, are safer compared to chemically synthesized antimicrobial drugs [10,11,12]. The components of Chinese herbal medicines are extremely complex, and the extraction and separation of their active antimicrobial ingredients can improve the efficiency of mildew inhibitors [13,14,15,16]. Some Chinese herbal medicines have identified antimicrobial monomers [17,18,19] and determined their chemical structures [20,21,22,23]. It has been reported that among Chinese herbal antimicrobial substances, phenolic compounds exhibit the strongest antimicrobial activity, followed by aldehydes, alcohols, ketones, esters, and hydrocarbons [24]. Studies have shown that the antimicrobial activity of Chinese herbal antimicrobial substances is closely related to the functional groups and structural arrangement of their active molecules. Phenolic compound molecules can bind to the negative charges in microbial cell walls, thereby disrupting the cell wall structure and interfering with microbial metabolism and growth [25]. In addition, the phenolic hydroxyl groups in phenolic compounds can also bind to proteins and DNA in microbial cell walls, further damaging the function and structure of microbial cells [26]. Therefore, it is crucial to conduct research on the antimildew properties and mechanisms of Chinese herbal phenolic compounds for bamboo mildew prevention.

However, to the best of our knowledge, there have been no reports of using phenolic substances to prevent mildew on sliced bamboo veneer. In this study, we aim to investigate the antimildew properties of phenolic substances derived from Chinese herbal medicines against mildews on sliced bamboo veneer and introduce new methods, such as the Oxford cup assay and the two-fold dilution method, to evaluate the antimildew properties of phenolic substances against bamboo mildews. The findings of this study can expand the application scope of phenolic compounds and are of great significance for solving the problem of bamboo materials being prone to mildew, enhancing the value and service life of bamboo products, and promoting the high-quality development of the bamboo industry.

## 2. Results and Discussion

### 2.1. Antimildew Activity of Three Traditional Chinese Medicine Phenolic Compounds against Bamboo Mildew

**Figure 1:**
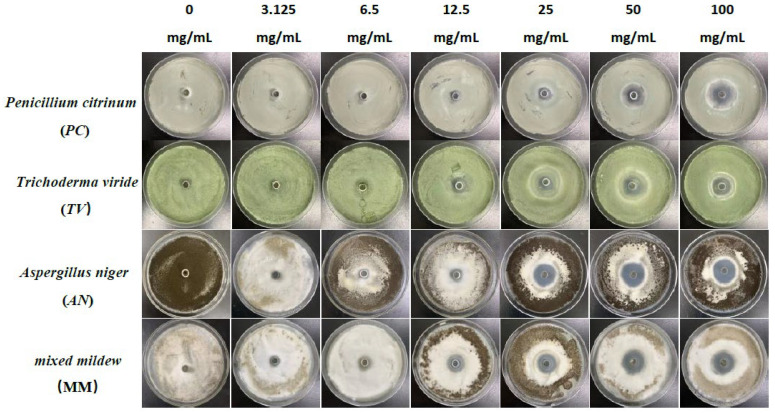
The growth inhibition effect of different concentrations of eugenol on bamboo mildews.

**Figure 2:**
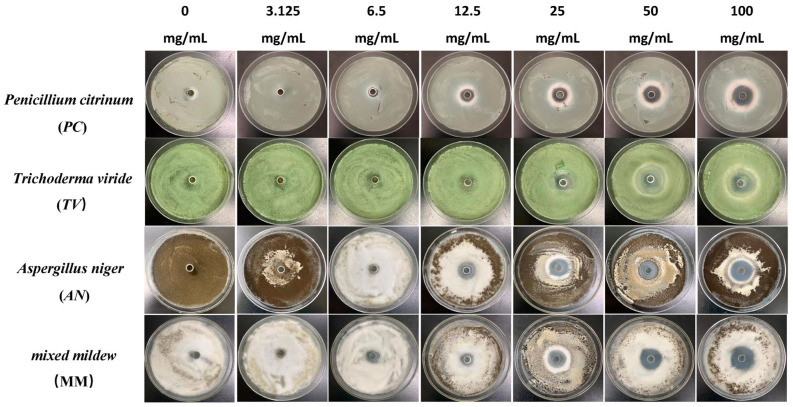
The growth inhibition effect of different concentrations of carvacrol on bamboo mildews.

**Figure 3:**
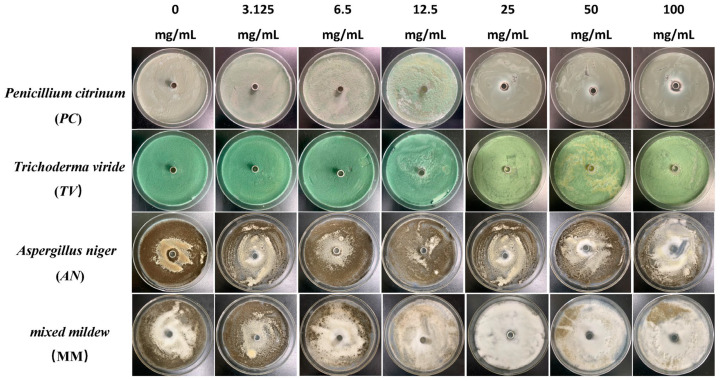
The growth inhibition effect of different concentrations of paeonol on bamboo mildews.

**Figure 4:**
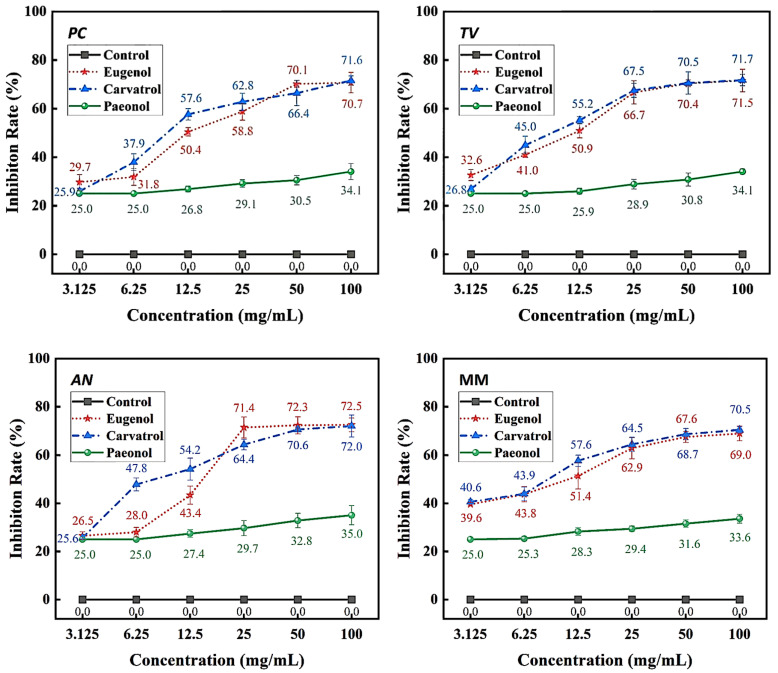
Inhibition rates of bamboo mildew by eugenol, carvacrol, and paeonol.

The Oxford cup method was used to study the effect of traditional Chinese medicine phenolic compounds, eugenol, carvacrol, and paeonol, on the inhibitory zone diameter against *Penicillium citrinum* (*PC*), *Trichoderma viride* (*TV*), *Aspergillus niger* (*AN*), and mixed mildews (MM) at six different concentration gradients. The inhibition rate was calculated based on the inhibitory zone diameter to characterize the strength of the antimildew ability of the three phenolic compounds. The experimental results are shown in Figure 1, Figure 2, Figure 3 and Figure 4.

According to the results in Figure 4, eugenol, carvacrol, and paeonol all exhibit inhibitory zones against *PC*, *TV*, *AN*, and MM, indicating their broad-spectrum antimildew properties.

Specifically, eugenol has a lower inhibition rate against *PC* at concentrations of 3.125 mg/mL and 6.25 mg/mL, but the inhibition rate significantly increases at concentrations of 25 mg/mL and 50 mg/mL, reaching 29.7% and 31.8%, respectively. As the concentration increases, the inhibition rate of eugenol continues to improve, with the highest inhibition rate of 70.7% at a concentration of 100 mg/mL. In contrast, carvacrol exhibits a certain inhibitory effect at a concentration of 3.125 mg/mL, and the inhibition rate continues to rise with increasing concentration. At a concentration of 50 mg/mL, the inhibition rate of carvacrol reaches 70.1%. At the highest concentration of 100 mg/mL, the inhibition rate further increases to 71.6%. However, paeonol shows a relatively weak inhibitory effect against *PC*, with an inhibition rate of only 34.1%, even at the highest concentration of 100 mg/mL. In summary, eugenol and carvacrol show significant inhibitory effects against *PC*, while paeonol demonstrates a relatively weaker inhibitory effect.

Furthermore, eugenol and carvacrol exhibit a significant upward trend in inhibition rate against *TV*. Eugenol’s inhibitory effect reaches 70.4% at a concentration of 50 mg/mL and 71.5% at 100 mg/mL; carvacrol’s inhibitory effect reaches 70.5% at a concentration of 50 mg/mL and increases to 71.7% at 100 mg/mL. However, paeonol ‘s inhibitory effect is relatively weak, with an inhibition rate of less than 35%, even at a concentration of 100 mg/mL. Therefore, overall, eugenol and carvacrol demonstrate better inhibitory effects against *TV* than paeonol.

Additionally, at low concentrations (3.125 mg/mL and 6.25 mg/mL), eugenol and carvacrol show lower inhibition rates against *AN*, but as the concentration increases, the inhibition rates significantly improve. Specifically, at concentrations of 50 mg/mL and 100 mg/mL, eugenol’s inhibition rates reach 72.3% and 72.5%, respectively, while carvacrol’s inhibition rates reach 70.6% and 72.0%, respectively. In comparison, paeonol exhibits a relatively lower increase in inhibition rate, demonstrating a significant inhibitory effect only at a concentration of 12.5 mg/mL, with the highest inhibition rate being only 35.0%.

For mixed fungi, eugenol and carvacrol exhibit lower inhibition rates at concentrations of 3.125 mg/mL and 6.25 mg/mL, but as the concentration increases, the inhibition rates significantly improve. At 50 mg/mL and 100 mg/mL, the inhibition rates of eugenol reach 67.6% and 69%, respectively. In contrast, carvacrol achieves a certain degree of inhibition even at lower concentrations, and as the concentration increases, the inhibition rate continues to rise, with the highest inhibition rate being 70.5%. Paeonol demonstrates a relatively lower inhibition rate against mixed fungi, especially at lower concentrations, but as the concentration increases, the inhibition rate gradually improves, with the highest inhibition rate being 33.6%. In summary, eugenol and carvacrol exhibit better inhibitory effects against mixed mildews than paeonol, and within a certain concentration range, the inhibitory effects significantly improve with increasing concentration.

The sensitivity of *PC*, *TV*, *AN*, and MM to these phenolic compounds varies, which may be related to the different biological pathways and mildew systems of the three phenolic compounds acting on different fungi [27].

### 2.2. Minimum Inhibitory Concentration (MIC) and Minimum Fungicidal Concentration (MFC) of Phenolic Compounds against Bamboo Mildew

The double dilution method was used to determine the MIC and MFC of eugenol, carvacrol, and paeonol against *Penicillium citrinum* (*PC*), *Trichoderma viride* (*TV*), *Aspergillus niger* (*AN*), and mixed mildews (MM). The lower the MIC and MFC values of the phenolic compounds, the stronger the antimildew effect. The experimental results are shown in Table 1.

Table 1 shows that different phenolic substances have a significant effect on the growth of the tested mildew strains. Carvacrol exhibited the strongest antimildew activity against all strains, with the lowest MIC and MFC values. It could inhibit and kill *AN* at a concentration of 0.98 mg/mL and inhibit the growth of all tested mildews at 1.56 mg/mL. At 1.76 mg/mL, it could kill all tested mildews, followed by eugenol. Paeonol had the lowest antimildew activity, with its MFC for *AN* being 2.19 times that of carvacrol. In theory, the MIC of MM should not be less than that of a single mildew to simultaneously inhibit the growth of the three mildews. However, the MFC of eugenol against MM was lower than that against *TV*. This suggests that under certain environmental conditions, the three mildew might compete, so the concentration of eugenol does not need to reach the maximum MFC of a single mildew to achieve simultaneous inhibition of the three mildews. As shown in Table 1, the MIC and MFC of eugenol, carvacrol, and paeonol for *PC*, *TV*, *AN*, and MM are different, indicating that different mildews have different tolerance to the agents, with *AN* having the smallest tolerance to the three phenolic compounds. From Table 1, the MIC and MFC values of eugenol, carvacrol, and paeonol against *PC*, *TV*, *AN*, and MM are between 0.98 and 2.34 mg/mL, and the ratio of MFC to MIC is between 1 and 1.23, reflecting strong antimildew performance. Paeonol did not show an obvious inhibition zone in the agar diffusion test for *PC*, *TV*, *AN*, and MM, but it exhibited a certain inhibitory activity when tested for MIC/MBC. This could be due to the different diffusion and distribution of paeonol in the agar medium between the two experiments. Therefore, the absence of a clear inhibition zone does not necessarily indicate the absence of antimildew activity, and similar phenomena have been observed in other studies on phenolic compounds [28].

### 2.3. Effects of Three Phenolic Compounds on the Mycelial Morphology of Bamboo Mildew

**Figure 5:**
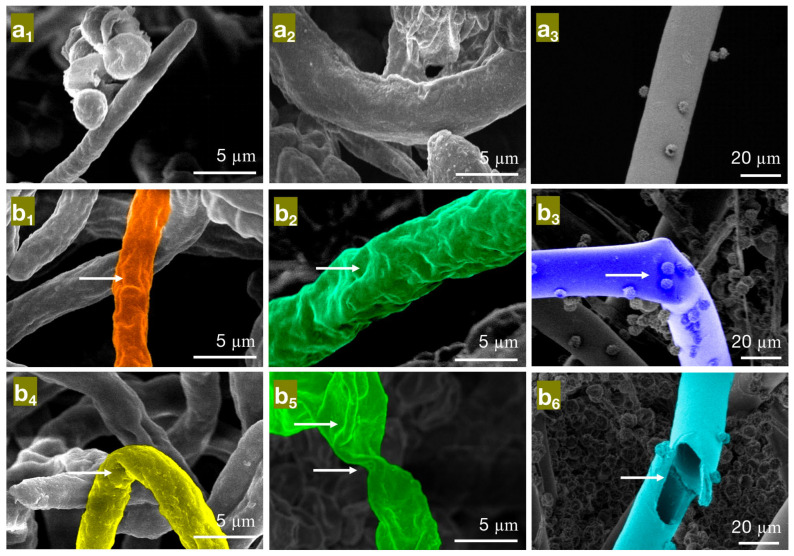
The effect of different concentrations of eugenol on the mycelial morphology of bamboo mildew ((**a_1_**–**a_3_**) are control groups of *PC*, *TV*, and *AN*, respectively; (**b_1_**–**b_3_**) are MIC treatment groups of *PC*, *TV*, and *AN*, respectively; (**b_4_**–**b_6_**) are MFC treatment groups of *PC*, *TV*, and *AN*, respectively).

**Figure 6:**
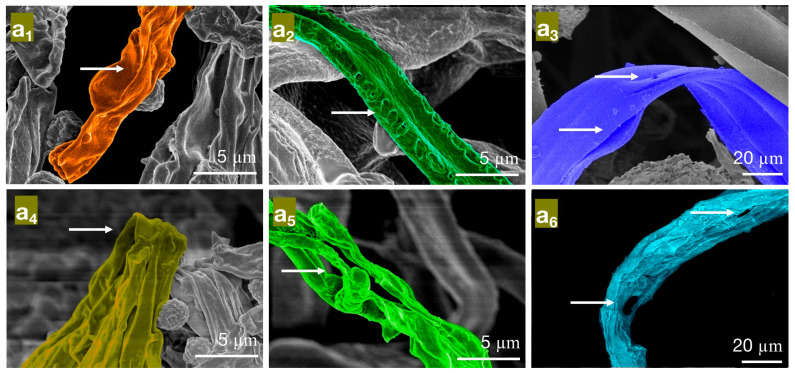
The effect of different concentrations of carvacrol on the mycelial morphology of bamboo mildew ((**a_1_**–**a_3_**) are MIC treatment groups of *PC*, *TV*, and *AN*, respectively; (**a_4_**–**a_6_**) are MFC treatment groups of *PC*, *TV*, and *AN*, respectively).

**Figure 7:**
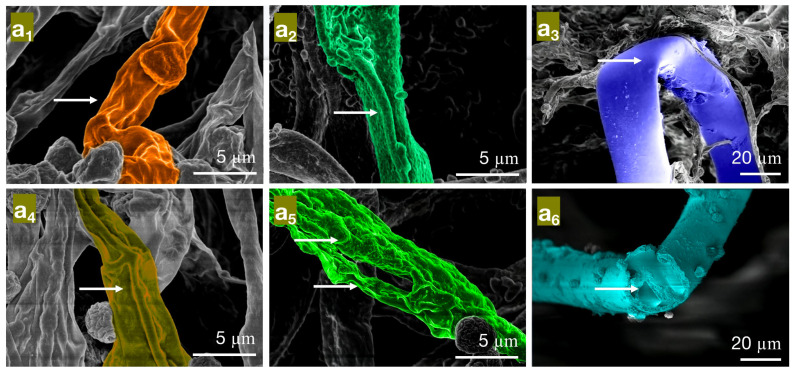
The effect of different concentrations of paeonol on the mycelial morphology of bamboo mildew ((**a_1_**–**a_3_**) are MIC treatment groups of *PC*, *TV*, and *AN*, respectively; (**a_4_**–**a_6_**) are MFC treatment groups of *PC*, *TV*, and *AN*, respectively).

Based on the results of the antimildew experiments, the MIC and MFC of the three phenolic compounds were used as indicators to treat *Penicillium citrinum* (*PC*), *Trichoderma viride* (*TV*), and *Aspergillus niger* (*AN*). The effects of these treatments on the mycelial morphology of bamboo mildew were observed using SEM, and the results are shown in Figure 5, Figure 6 and Figure 7.

As seen in Figure 5, in the control group (Figure 5a_1_–a_3_), the hyphae of *PC*, *TV*, and *AN* mildew appear linear, full, and regular in shape, robust, and structurally intact. However, in the minimum inhibitory concentration (MIC) treatment group of eugenol, the hyphae of *PC* (Figure 5b_1_) exhibit surface unevenness, rough hyphae, and irregular thickness; *TV* (Figure 5b_2_) have deformed, collapsed hyphae with wrinkles; *AN* (Figure 5b_3_) show irregularly twisted and bent hyphae. These observations may be due to eugenol affecting the substance transport within and outside the mildew cells, leading to a decrease in intracellular material levels or interfering with mildew cell wall metabolism and synthesis, causing the observed morphological changes [29]. In the MFC treatment group of eugenol, the hyphae of *PC* (Figure 5b_4_) are not only shriveled and rough but also exhibit hyphal bending; *TV* (Figure 5b_5_) have severely shriveled and distorted hyphae; *AN* (Figure 5b_6_) display severely twisted hyphae with ruptures and numerous holes. These observations further confirm that eugenol can damage the mildew cell wall, causing severe leakage of intracellular substances. In summary, eugenol has a significantly destructive effect on the shape and structural integrity of the hyphae of bamboo mildew, and the degree of destruction is directly proportional to the concentration of eugenol. Moreover, at MFC concentrations, eugenol can severely affect the morphology of mildew hyphae, destroy their structure, and, thus, achieve a fungicidal effect.

In the MIC treatment group of carvacrol, the hyphae of *PC* (Figure 6a_1_) exhibit evident shriveling, hyphal shrinkage, and uneven thickness; *TV* (Figure 6a_2_) have shriveled, softened hyphae with a rough surface, increased surface area, and a dissolving trend; *AN* (Figure 6a_3_) display flattened hyphae with leaking contents, different degrees of bending, and hyphal cracks. In the MFC treatment group of carvacrol, the hyphae of *PC* (Figure 6a_4_) completely collapse on the mycelial cake surface, losing support; *TV* (Figure 6a_5_) have twisted, ruptured, and contracted hyphae; *AN* (Figure 6a_6_) display filamentous cracks, numerous holes, and loss of contents. Based on these observations, carvacrol may have a toxic effect on the cell membrane structure of bamboo mildew, causing damage to the hyphal cell membrane and cell wall or leading to the loss of membrane fluidity and increased cell wall rigidity, ultimately causing membrane lysis [30,31]. In summary, carvacrol has a significantly destructive effect on the shape and structural integrity of the hyphae of bamboo mildew, and the degree of destruction is positively correlated with the carvacrol treatment concentration. Additionally, at MFC concentrations, carvacrol can severely damage the morphology of mildew hyphae, thus achieving a fungicidal effect.

In the minimum inhibitory concentration (MIC) treatment group of paeonol, the hyphae of *Penicillium citrinum PC* (Figure 7a_1_) show reduced contents, shriveling, hyphal shrinkage, and uneven thickness; *Trichoderma viride* (*TV*) (Figure 7a_2_) exhibit an increased roughness on the hyphal surface, shriveled and wrinkled hyphae; *Aspergillus niger AN* (Figure 7a_3_) display softened hyphae with various degrees of bending, as well as creases and cracks on the hyphae. In the MFC treatment group of paeonol, the hyphae of *PC* (Figure 7a_4_) further shrivel and contract; *TV* (Figure 7a_5_) show a dissolving trend, with thicker, more transparent hyphae and the appearance of holes; *AN* (Figure 7a_6_) exhibit broken, severely ruptured hyphae with no visible contents inside. Based on these observations, paeonol may have an impact on the cell membrane structure of bamboo mildew, affecting the transport of substances inside and outside the cells, possibly causing damage to the hyphal cell membrane and cell wall [32], and ultimately resulting in hyphal lysis or loss of resilience. In summary, paeonol has a significantly destructive effect on the shape and structural integrity of the hyphae of bamboo mildew, and the degree of destruction is directly proportional to the paeonol concentration. The electron microscopy observations are consistent with the minimum inhibitory and fungicidal experimental results, further confirming that paeonol has an inhibitory performance and can severely damage the morphology of mildew hyphae at MFC concentrations, thus achieving a fungicidal effect.

### 2.4. Effects of Eugenol, Carvacrol, and Paeonol on the Extracellular Fluid pH of Bamboo Mildews

**Figure 8:**
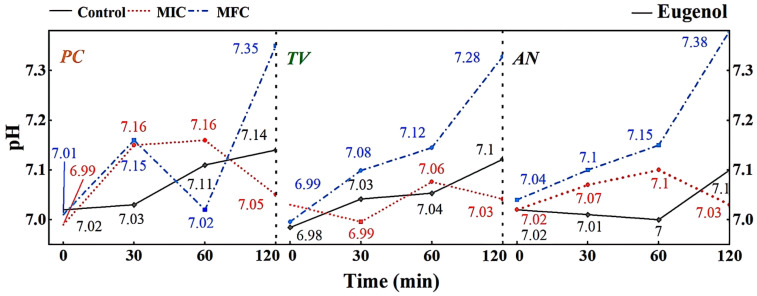
Effect of eugenol on pH value of extracellular fluid of bamboo fungus (Control: control group; MIC: MIC treated group; MFC: MFC treated group).

A micro pH meter was used to measure the changes in extracellular fluid pH of *PC*, *TV*, and *AN* treated with different concentrations of eugenol over time, as shown in Figure 8.

The extracellular fluid pH is a critical factor influencing cell DNA transcription, protein synthesis, and enzyme activity. Compared to other eukaryotes, mildew cells have a more robust pH regulation and H+ transport mechanism, allowing them to adapt to more extreme growth and infection environments [33]. As shown in Figure 8, different concentrations of eugenol can affect the extracellular pH of bamboo mildews. In the control group, the extracellular pH of bamboo mildew changed very little or remained unchanged. Within the 0–60 min treatment range, the extracellular fluid pH of the treated mildew groups showed an overall increasing trend, indicating leakage of intracellular metal ions and other alkaline components to the extracellular fluid, leading to cell acidification, intracellular H+ accumulation, and irreversible damage to intracellular physiological and biochemical processes. After 60 min, the extracellular fluid pH of mildew strains treated with MIC began to decline gradually, which may be due to self-regulation by the cell in an attempt to maintain the balance of intracellular and extracellular fluid environments by secreting acid. The extracellular fluid pH of mildew strains treated with MFC showed a more significant increase, possibly due to severe cell membrane damage in bamboo mildews, rendering the cell unable to restore acid secretion balance. As shown in Figure 8, the changes in extracellular fluid pH of bamboo mildew varied among different strains, and the trends of extracellular pH changes in bamboo mildew treated with different concentrations of eugenol also varied. The higher the concentration of eugenol, the greater its influence on the regulation of extracellular pH in bamboo mildews. Therefore, the trend of changes in the extracellular pH of bamboo mildew cells treated with eugenol may be related to the strain type and eugenol treatment concentration. Eugenol may exert its inhibitory or fungicidal effect on mildew by disrupting cell membrane permeability, which is consistent with previous research findings [34,35]. This may be because phenolic compounds can denature proteins in the cell wall, proteins are key components responsible for the transport of substances in the cell wall, and their denaturation can cause disorder in cell wall permeability.

**Figure 9:**
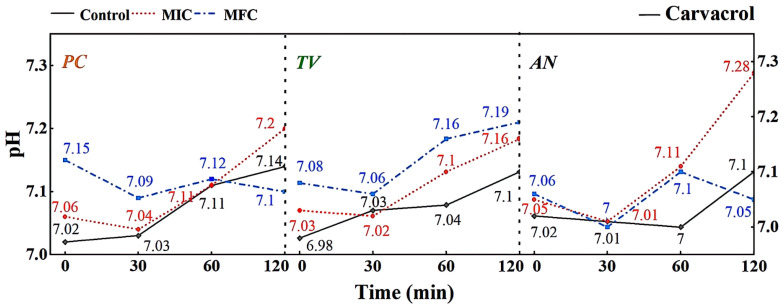
Effect of carvacrol on pH value of extracellular fluid of bamboo fungus (Control: control group; MIC: MIC treated group; MFC: MFC treated group).

According to Figure 9, different concentrations of carvacrol can significantly affect the extracellular pH of bamboo mildews. Within the 0–30 min treatment range, the extracellular fluid pH of the treated mildew groups showed an overall decreasing trend, indicating proton efflux from the cells, leading to acidification of the extracellular fluid. This may be due to structural damage of the proton ATPase protein in bamboo mildew caused by carvacrol, resulting in the loss of the ability to maintain proton concentration gradients [36]. Within the 30–120 min treatment range, the extracellular pH of the MIC treatment group showed an overall increasing trend, indicating leakage of intracellular metal ions and other alkaline components to the extracellular fluid. This may be because phenolic compounds can bind to lipid molecules on the mildew membrane, causing structural changes in the membrane and, thus, affecting the function of ion channels. Another possible mechanism is that phenolic compounds can affect ATP production and metabolism within mildew cells, thereby affecting the function of proton pumps and channels. The extracellular pH of the MFC-treated group showed an initial upward trend followed by a downward trend. This may be because as the concentration of carvacrol increases, the degree of cell membrane damage increases, resulting in more acidic substances, such as nucleic acids, phospholipids, and cell fluid flowing out, causing pH to decrease again. Therefore, it can be inferred that carvacrol disrupts the intracellular environment by damaging the cell membrane structure, thereby affecting the metabolism and growth of fungi. It is worth noting that compared with eugenol, carvacrol has a different pattern of extracellular pH changes in bamboo mildew. This may suggest that eugenol and carvacrol target different sites on the cell membrane and cell wall of bamboo mildew fungi.

**Figure 10:**
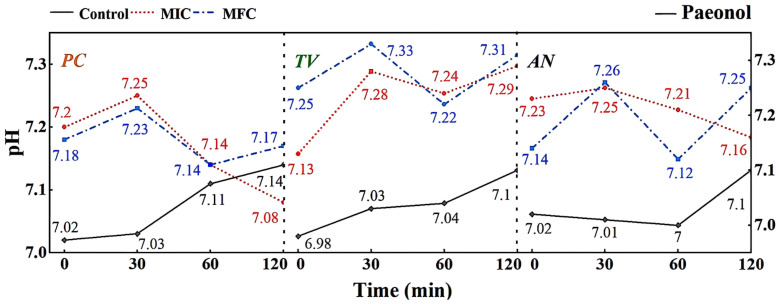
Effect of paeonol on pH value of extracellular fluid of bamboo fungus (Control: control group; MIC: MIC treated group; MFC: MFC treated group).

According to the results shown in Figure 10, it can be observed that different concentrations of gambierol can affect the extracellular pH of bamboo mildew. Within the treatment range of 0–30 min, the pH of the extracellular fluid of the treated strains generally showed an upward trend, indicating that alkaline components, such as metal ions, leaked out of the cells. This may be due to the fact that the gambierol treatment destroyed the membrane structure of bamboo mildew, affecting the function of ion channels. After 30 min, the extracellular fluid pH of the treated strains showed a downward trend, possibly because the cells began to release protons outside to maintain the balance of the intra- and extracellular environment. Within the treatment range of 60–120 min, the extracellular fluid pH of the strains treated with MFC increased again. Combined with the results of electron microscopy, it was found that the structure of the treated mildew hyphae was severely damaged, and intracellular substances were lost to a large extent. It was suggested that gambierol might further damage the membrane and wall structures of bamboo mildew, causing alkaline substances, such as cytoplasmic matrix, mitochondrial matrix, vacuoles, nucleoli, amino acids, nucleotides, calcium ions, and sodium ions, to leak out of the cells.

### 2.5. Effect of Carvacrol on the Cell Structure of Bamboo Mildew

**Figure 11:**
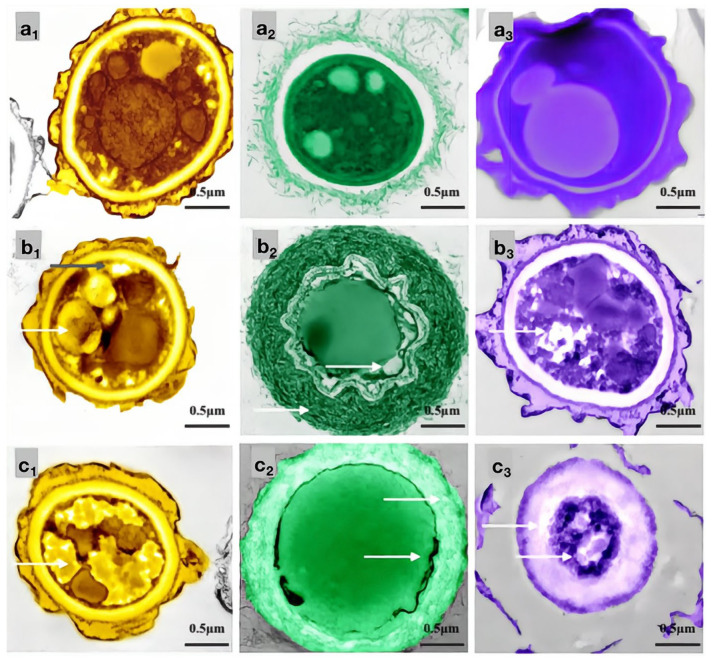
Effects of carvacrol on cellular structures of bamboo mildew ((**a_1_**–**a_3_**) were the *PC* control group, MIC treatment group, and MFC treatment group, respectively; (**b_1_**–**b_3_**) were the *TV,* respectively, control group, MIC treatment group, and MFC treatment group; (**c_1_**–**c_3_**) were the *AN* control group, MIC treatment group, and MFC treatment group, respectively).

In this study, the inhibitory effects of carvacrol, eugenol, and paeonol on common bamboo mildew, including *Penicillium citrinum* (*PC*), *Trichoderma viride* (*TV*), *Aspergillus niger* (*AN*), and mixed mildews (MM), were systematically studied using the Oxford cup method and the doubling dilution method. The results showed that the inhibitory effect of carvacrol on MM was higher than that of eugenol and paeonol. Carvacrol had the lowest MIC and MFC values for *PC*, *AN*, and *TV*. SEM observations indicated that carvacrol caused the most severe structural damage to the mycelium of *PC*, *AN*, and *TV*. Thus, carvacrol exhibited the strongest antimicrobial effect on bamboo mildews. Using the MIC and MFC of carvacrol as indicators, the inhibitory effects of carvacrol on *PC*, *TV*, and *AN* were evaluated. Then, transmission electron microscopy (TEM) was used to observe the effect of carvacrol on the cell structure of bamboo mildews and further clarify its antimicrobial mechanism. The experimental results are shown in Figure 11.

As shown in Figure 11, the mycelial cells in the control group (Figure 11a_1_–c_1_) were intact, and the organelles were evenly distributed. The protoplasm was densely distributed in the cells, and the cell walls had uniform thickness, indicating normal growth. In the MIC treatment group, the cell membrane of *PC* (Figure 11a_2_) became transparent, and the content of intracellular light-colored liquid increased. The morphology of the *TV* mycelium (Figure 11b_2_) was significantly deformed; the organelles began to disappear, the cell wall was shriveled, and the cell membrane was distributed in a crescent shape, with flagella visibly shrinking and clustering around the cell membrane. The cell wall of *AN* (Figure 11c_2_) thickened significantly, and the extracellular substance became thin and transparent. The membrane of the organelles collapsed, the boundary was not clear, and a light-colored liquid appeared in the cells. In the MFC treatment group, the cell wall of *PC* (Figure 11a_3_) thickened significantly, the light-colored liquid aggregated in the cells, and the boundary of the cytoplasm disappeared. The cell wall and membrane of *TV* (Figure 11b_3_) dissolved severely, and the cell wall became thicker and more transparent. The cell membrane was almost invisible, and the organelles and cytoplasm were almost absent in the cells. The cell wall of *AN* (Figure 11c_3_) became severely thick and loose, almost dissolving and becoming transparent, and the boundary of the cytoplasm was almost indistinguishable. The color in the cells became darker, and the amount of light-colored liquid increased further. These results indicated that carvacrol at various concentrations could significantly destroy the cell structure of bamboo mildews, and the degree of damage increased with increasing concentration. *AN*, *TV*, and *PC* are multicellular mildew with cell walls that act as barriers against mechanical and osmotic damage, and cell membranes are crucial components for maintaining cell balance and performing substance exchange and energy transfer. Therefore, when the integrity of the cell membrane is compromised, self-digestion of the mildew occurs, and its life activity is inhibited, which further supports the mechanism of carvacrol in achieving its antimicrobial effect by damaging the cell membrane and cell wall structure of mildew.

### 2.6. Antimildew Effect of Different Concentrations of Carvacrol on Sliced Bamboo Veneer

**Figure 12:**
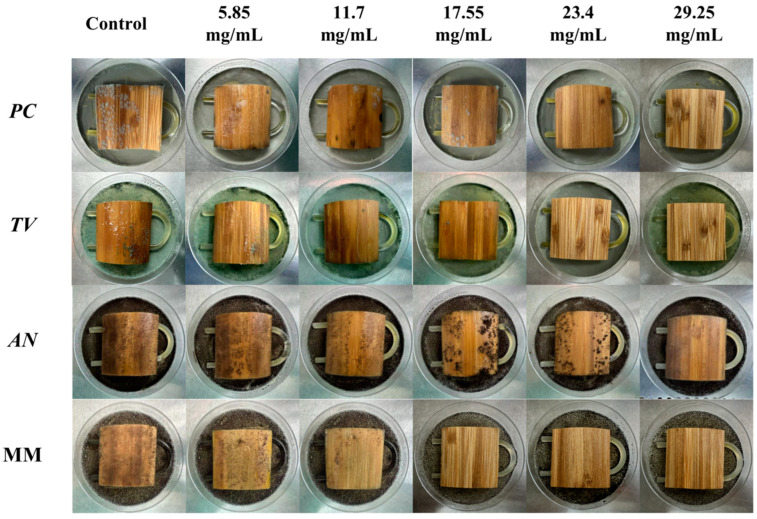
Mildew-grade of sliced bamboo veneers were treated with carvacrol at different concentrations on the 28th day.

**Figure 13:**
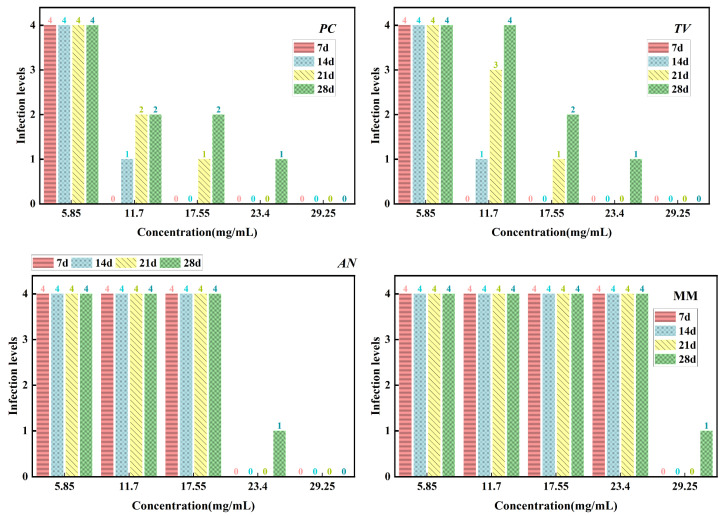
Mildew-grade of sliced bamboo veneers treated with carvacrol at different concentrations on the 28th day.

According to the evaluation criteria for antimildew properties of engineered wood, five different concentrations of carvacrol were selected for testing: 3 times (5.85 mg/mL), six times (11.7 mg/mL), nine times (17.55 mg/mL), 12 times (23.4 mg/mL), and 15 times (29.25 mg/mL) the minimum fungicidal concentration (MFC), respectively, to treat sliced bamboo veneers for antimildew purposes. The results obtained on the 28th day of the antimildew test are shown in Figure 12, and the antimildew grades calculated according to the relevant evaluation standards are shown in Figure 13.

From Figure 13, it can be seen that on the 7th day of the antimildew experiment, the surface mildew growth grade of *TV* on sliced bamboo treated with carvacrol solutions of five concentrations (5.85 mg/mL, 11.7 mg/mL, 17.55 mg/mL, 23.4 mg/mL, and 29.25 mg/mL) was 4, 4, 4, 0, and 0, respectively. This indicates that carvacrol solutions with concentrations of 5.85 mg/mL, 11.7 mg/mL, and 17.55 mg/mL did not exhibit antimildew effects against *AN* on sliced bamboo on the 7th day of the experiment, while the other two concentrations showed significant antimildew effects. On the 28th day, the surface mildew growth grade of *AN* on sliced bamboo treated with carvacrol solutions of five concentrations was 4, 4, 4, 1, and 0, respectively, indicating that carvacrol solutions with concentrations of 5.85 mg/mL, 11.7 mg/mL, and 17.55 mg/mL did not exhibit antimildew effects or showed significantly reduced antimildew effects against *AN*, while carvacrol solutions with concentrations of 23.4 mg/mL and 29.25 mg/mL met the antimildew standards.

From Figure 13, it can be observed that the surface mildew growth grade of MM on sliced bamboo treated with carvacrol solutions of five concentrations (5.85 mg/mL, 11.7 mg/mL, 17.55 mg/mL, 23.4 mg/mL, and 29.25 mg/mL) was 4, 4, 4, 4, and 0, respectively. This indicates that carvacrol solutions with concentrations of 5.85 mg/mL, 11.7 mg/mL, 17.55 mg/mL, and 23.4 mg/mL did not exhibit effective antimildew effects against MM on sliced bamboo on the 7th day of the experiment, while only the concentration of 29.25 mg/mL showed significant antimildew effects. On the 28th day of the antimildew experiment, the surface mildew growth grade of MM on sliced bamboo treated with carvacrol solutions of five concentrations was 4, 4, 4, 4, and 1, respectively, and only the concentration of 29.25 mg/mL met the antimildew standards, with a surface mildew growth grade of 1. In conclusion, the antimildew efficacy of carvacrol against MM is relatively weak.

In summary, on the 28th day of the mildew prevention experiment, the carvacrol solution with a concentration of 29.25 mg/mL can achieve a mildew growth grade of 1 on the surface of the specimens of sliced bamboo veneers against MM, indicating that this concentration has a good mildew prevention effect against MM; while for the other three mildews, the inhibition effect is more significant, and the carvacrol solution with a concentration of 29.25 mg/mL can achieve a mildew growth grade of 0 on the surface of the specimens of sliced bamboo veneers against *PC*, *TV*, and *AN*, respectively. Therefore, the concentration of 29.25 mg/mL carvacrol can be used as the optimal impregnation concentration for the mildew prevention treatment of sliced bamboo.

**Figure 14:**
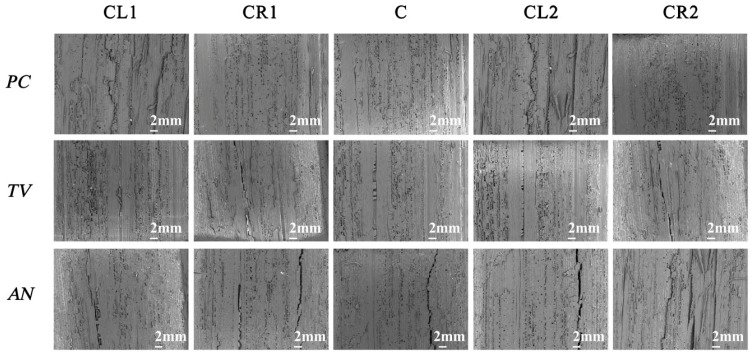
Image of *PC*, *TV*, and *AN* after 28-day antimildew treatment with 29.25 mg/mL of carvacrol, observed under an electron microscope. CL1, CR1, C, CL2, and CR2 refer to the upper-left, upper-right, center, lower-left, and lower-right parts of the sliced bamboo veneers taken for photography.

According to the “Evaluation of Antimicrobial Properties of Wood-Based Panels” standard, the sliced bamboo samples without visible mildew hyphae growth were selected from the table and observed immediately under a low magnification (50×) biological optical microscope. Figure 14 shows the electron microscopy observation of the sliced bamboo veneers.

According to the experimental results shown in Figure 14, at a concentration of 29.25 mg/mL of carvacrol, the antimildew effect on the orange mildew (*PC*), green mildew (*TV*), and black mildew (*AN*) of the sliced bamboo veneers reached the strong antimildew level (level 0).

## 3. Materials and Methods

### 3.1. Materials

#### 3.1.1. Sliced Bamboo Veneer

Large-format bamboo veneers, sliced to dimensions of 2000 mm × 450 mm (length × width) and a thickness of 0.55 mm, were sourced from Hangzhou Senrui Bamboo and Wood Industry Co., Ltd. (Hangzhou, China). The veneers had a moisture content of 8–10% without non-woven fabric on the back. To suit the experimental process, the large-format sliced bamboo veneers were cut into a size specification of 35 mm × 35 mm.

#### 3.1.2. Main Reagents

Eugenol (4-allyl-2-methoxyphenol, 99%), carvacrol (2-methyl-5-isopropylphenol, 99%), and paeonol (2-hydroxy-4-methoxybenzophenone, 99%) were purchased from Hongli Biotechnology (Cambridge, MA, USA); agar powder and glucose were of analytical grade and purchased from Sinopharm Chemical Reagent Co., Ltd. (Beijing, China); Tween-80 (T) was of chemically pure grade and purchased from Shanghai Lingfeng Chemical Reagent Co., Ltd. (Shanghai, China); deionized water (prepared in-laboratory).

#### 3.1.3. Mildews

*Penicillium citrinum* (*PC*), *Trichoderma viride* (*TV*), *Aspergillus niger* (*AN*), and mixed mildews (MM, a mixture of *PC*, *TV*, and *AN* in equal proportions). All of them were obtained from the microbial research laboratory of Zhejiang A&F University and isolated directly from the natural moldy bamboo material. After purified and cultured, and identified by repeated inoculation tests and microscopic examination.

### 3.2. Methods

#### 3.2.1. Preparation of Potato Dextrose Agar (PDA) Medium

To prepare the PDA medium, freshly peeled potatoes (400 g) were washed, chopped, and boiled in deionized water for 30 min. The filtrate was collected through a double-layer cheesecloth. Next, glucose (40 g) and agar (46 g) were added to the filtrate, which was then diluted to 2000 mL with water. The mixture was stirred until the agar was completely dissolved. Finally, the medium was poured into conical flasks, sealed with high-temperature sealing film, and autoclaved at 121 °C and 0.1 MPa for 1 h. After sterilization, the medium was poured out to make potato dextrose agar plates for subsequent mildew culture.

#### 3.2.2. Preparation of Fungal Suspension

To prepare the fungal suspension, deionized water and small glass beads were placed in a wide-mouthed bottle and autoclaved at 121 °C and 0.1 MPa for 1 h. Inoculation was carried out at a clean biological bench (sterile environment). Mycelium and spores of the test mildew were picked with an inoculation needle and placed in the autoclaved wide-mouthed bottle. The suspension was shaken for 10 to 15 min to obtain a mildew suspension for inoculation.

#### 3.2.3. Preparation of Phenolic Compounds

To prepare a stock solution, a 10 g sample of the reagent was pipetted and mixed with 2 mL of Tween-80 (2% of the total volume) and deionized water. The mixture was transferred to a 100 mL volumetric flask and diluted to 100 mL with deionized water to obtain a 100 mg/mL stock solution. Two-fold serial dilutions were performed to obtain samples with concentrations of 100 mg/mL, 50 mg/mL, 25 mg/mL, 12.5 mg/mL, 6.25 mg/mL, and 3.125 mg/mL.

#### 3.2.4. Antimildew Properties of Three Phenolic Compounds

The antimildew properties of three Chinese herbal medicine phenolic compounds on bamboo mildew were measured using the Oxford cup method. The culture medium plate was coated with 80 µL of mildew suspension, and the drug amount was 80 µL/cup. The samples were cultured in a constant temperature incubator at 28 °C and 85% ± 5% humidity. After 48 h of cultivation, the diameter of the inhibition zone was measured using the cross method, the mean value of each experimental result was taken from three parallel experiments, and the inhibition rate was calculated according to Formula (1). A 2% Tween-80 aqueous solution was used as a control, and a blank experiment was carried out.

(1) $$E\;=\frac{{\;D}_{1}-D_{0}}{D_{0}}\times 100\%$$

where E is the inhibition rate (%), D_1_ is the average infection ratio of extracted, specimens and D_0_ is the diameter of the Oxford cup. The inhibition rate of a specific group of specimens is defined as the mean value of their E values against the three individual mildews and the mildew mix.

#### 3.2.5. Minimum Inhibitory Concentration (MIC) and Minimum Fungicidal Concentration (MFC)

The MIC and MFC of the phenolic compounds were tested using the twofold dilution method. Specifically, 2 mL of phenolic compounds, Tween-80, and culture medium were mixed to prepare 100 mL of culture medium with varying concentrations. Subsequently, 80 µL of mildew suspension was evenly spread on the surface of the culture dish, which was then sealed with sterile sealing film and placed in a constant temperature incubator at 28 °C and 85 ± 5% humidity. After 48 h of observation, the lowest concentration of the drug group with no mildew growth was identified as the MIC. Following 7 days of further incubation, the lowest concentration of the drug group with no mildew growth was considered the MFC. A 2% Tween-80 aqueous solution was used as a blank control.

#### 3.2.6. Effect of Three Phenolic Compounds on the Morphology of Bamboo Mildew Hyphae

The effect of eugenol on the morphology of the hyphae of three bamboo mildew was observed using a scanning electron microscope (SEM) SU8010. Fungal cakes with a diameter of 8.0 mm were removed from the vigorously growing mildew culture medium plates after 7 d of cultivation and placed on the surface of the eugenol culture medium plates with concentrations of 0, MIC, and MFC. The samples were then cultivated at a temperature of 28 °C and 85 ± 5% humidity. After 4 d, the mildew cakes were harvested and prepared for electron microscopy.

The samples were fixed overnight with 2.5% glutaraldehyde at 4 °C, then washed three times with phosphate-buffered saline (PBS, pH = 7.0), 15 min each time. The samples were washed three more times with PBS, 15 min each time, then dehydrated in a series of ethanol gradient solutions (30%, 50%, 70%, 80%, 90%, and 95%), 15 min each time, followed by dehydration in anhydrous ethanol for 20 min. The final treated samples were vacuum freeze-dried and gold-sputtered, and the hyphal surface morphology was observed using a scanning electron microscope.

#### 3.2.7. Effect of Three Phenolic Compounds on the Extracellular Fluid pH of Bamboo Mildew

To evaluate the impact of three phenolic compounds on the extracellular fluid pH of bamboo mildew, we utilized a micro pH meter (PHS-3C). We collected fungal spores that had been cultured for approximately 7 days and washed them thrice with phosphate-buffered saline before suspending them in a buffer solution. The spore suspension was treated with varying concentrations (0, MIC, and MFC) of phenolic samples for 0 min, 30 min, 60 min, and 120 min. Following treatment, we measured the pH of the extracellular fluid of 5 mL of the spore suspension. Each experiment was conducted three times, with a 2% Tween-80 deionized water solution serving as a blank control.3.2.8 Effect of carvacrol on the cellular structure of bamboo mildew.

The microstructure of the treated mildew cells was observed using a transmission electron microscope (TEM, JEM-1200, Tokyo, Japan). For the carvacrol samples, the preliminary treatment method was the same as in 3.2.6. The difference was that after the ethanol series gradient dehydration treatment, the samples were transitioned to pure acetone treatment for 20 min. Then, the samples were treated with a mixture of embedding agent and acetone (*v*/*v* = 1/1) for 1 h and with a mixture of embedding agent and acetone (*v*/*v* = 3/1) for 3 h. The samples were then treated with pure embedding agent overnight, and the infiltrated samples were embedded and heated overnight at 70 °C to obtain well-embedded samples. The samples were sectioned using an ultramicrotome to obtain 70–90 nm sections, which were stained with lead citrate and 50% saturated uranyl acetate in ethanol for 5–10 min, respectively. The samples were then air-dried and observed under a transmission electron microscope for the microstructure of the mildew cells.

#### 3.2.8. Indoor Antimildew Test of Carvacrol-Treated Sliced Bamboo Veneer

Five different concentrations of carvacrol (5.85 mg/mL, 11.7 mg/mL, 17.55 mg/mL, 23.4 mg/mL, and 29.25 mg/mL) were selected, and a processing technology with a sliced bamboo size of 350 mm × 350 mm and an immersion time of 2.5 h was used to carry out antimildew treatment on the sliced bamboo veneers, and the antimildew experiment was carried out according to the Chinese national standard “Test Method for Antimildew and Antistain Efficacy of Fungicides on Wood Mildew and Stain Mildew (GB/T18261-2013) [37] and “Evaluation of Antimildew Performance of Wood-based Panels” (LY/2230-2013) [38]. The infection of sliced bamboo veneers by *Penicillium citrinum* (*PC*), *Trichoderma viride* (*TV*), *Aspergillus niger* (*AN*), and mixed mildews (MM) was observed and analyzed every other day. The antimildew grading value was the average value of the mildew infection area of the two test groups.

## 4. Conclusions

In this study, the inhibitory effects of three phenolic compounds, namely eugenol, carvacrol, and paeonol, on common bamboo mildew species were systematically studied. The results showed that eugenol and carvacrol exhibited significantly better antimicrobial activity than paeonol. At a concentration of 25 mg/mL, eugenol and carvacrol exhibited an inhibition rate of over 50% against Penicillium citrinum, Trichoderma viride, Aspergillus niger, and mixed mildews. The three phenolic compounds can significantly disrupt the shape, morphology, and structural integrity of mildew hyphae. Carvacrol exhibited the lowest MIC and MFC values, indicating strong antimicrobial activity. Moreover, SEM and TEM technologies showed that carvacrol could destroy the cell wall and cell membrane of bamboo mildew, affecting its morphology and structure, and damaging the physiological and biochemical processes inside the cell, thereby achieving the effect of inhibiting or killing bamboo mildew. The antimildew performance of carvacrol-treated sliced bamboo veneers against mildew was also evaluated, indicating that carvacrol has the potential as a natural antimicrobial agent for bamboo products.

## Figures and Tables

**Table 1:** MIC and MFC of Eugenol, Carvacrol and Paeonol against bamboo mildew.

Phenolic Compounds	*PC*		*TV*		*AN*		MM	
	MIC mg/mL	MFC mg/mL	MIC mg/mL	MFC mg/mL	MIC mg/mL	MFC mg/mL	MIC mg/mL	MFC mg/mL
Eugenol	1.56	1.95	1.95	2.15	1.37	1.56	1.56	1.95
Carvacrol	1.56	1.76	1.36	1.56	0.98	0.98	1.56	1.76
Paeonol	2.15	2.34	1.95	2.15	1.95	2.15	2.15	2.34

## Data Availability

Not applicable.
